# Social interdependencies: the deep evolutionary roots of morality and normativity

**DOI:** 10.1007/s10539-026-10013-z

**Published:** 2026-03-06

**Authors:** Carel P. van Schaik, Rahel K. Brügger, Judith M. Burkart

**Affiliations:** 1 Department of Evolutionary Anthropology, University of Zurich, Winterthurerstrasse 190, 8057 Zurich, Switzerland; 2 Center for the Interdisciplinary Study of Language Evolution (ISLE), University of Zurich, Affolternstrasse 56, 8050 Zurich, Switzerland; 3 Comparative Socioecology Group, Max Planck Institute of Animal Behavior, Bücklestrasse 5A, E78467 Constance, Germany

**Keywords:** Animal morality, Animal normativity, Evolution of morality, Evolution of normativity

## Abstract

We propose that minimal morality (experiencing “ought-thoughts”) and minimal normativity (having expectations or “standards”) historically emerged in the context of parental care among animals. Subsequently, in animals that formed stable, personalized societies, the moral motivation was functionally extended to enable interdependent social relationships (bonds, with kin, but also with non-kin as same-sex social or pair bonds), whereas the presence of normative expectations for the partner’s behavior in social bonds emerged concomitantly because the ever-present risk of shirking requires evaluation of the partner’s social actions. This social evaluation is extended toward the actions of other group members whenever individuals can switch to other bonded partners or allies. The spectrum of responses to partners’ actions can range from shunning to punishment, depending on the strength of the fitness-augmentation effects of bonds or alliances. We do not expect individuals to socially evaluate the actions of non-group members, except in dispersal situations. This functional hypothesis for the evolution of morality and normativity is supported by extensive behavioral and physiological evidence from various social animals. In sum, we argue that morality and normativity are fundamentally social, both serving to sustain dyadic relationships of interdependence that are inherently threatened by free riding. Human morality and normativity are an extension of this basic dyadic version, by involving group-level interdependencies, and presumably also by involving an awareness of the same motivations and social evaluations in others, which, through language-based negotiation and reflection, permits the explicit formulation of moral prescriptions, which may therefore vary culturally.

## Introduction

Morality and normativity are both essential characteristics of what it means to be human, and have traditionally been considered uniquely human, for instance by being linked to religion (Bloom 2012). In recent decades, it has become increasingly clear that some of the behavioral and cognitive elements of the phenomena in humans—often referred to as building blocks—are shared with other species (de Waal et al. 2006; de Waal 2014; Brosnan and de Waal 2014; Fitzpatrick 2017; Burkart et al. 2018; Kappeler et al. 2019; Westra et al. 2024; Andrews et al. 2024). Most of these studies have focused on behaviors that are interesting from the perspective of human morality, such as peaceful interventions or mediation in conflicts (Rudolf von Rohr et al. 2012; de Waal 2014), joint protests against infanticide (Rudolf von Rohr et al. 2015) and gratuitous aggression against females (de Waal and Aureli 1996), or even high-level concepts such as inequity aversion (including notions like fairness: Brosnan and de Waal 2014). The focus in these comparisons tends to be on actors as uninvolved bystanders protecting the weak or maintaining social peace or even upholding a general norm.

Important as these studies are, by highlighting the behavior of non-involved, third-party bystanders rather than the individuals directly involved as first parties (“one-on-one” morality in de Waal 2014), they focus on features that are clearly derived. However, if we are to identify the evolutionary origins of morality among animals, we must also focus on situations in which first parties, those directly interested in behavioral outcomes, show evidence of morality and normativity. Our goal here is to identify the fundamental contexts in which morality and normativity first arose. We acknowledge that human morality and normativity extend way beyond their origins in non-human animals. Our human socio-cognitive abilities allow for a multifold complexity of cooperative acts, and language makes the establishment of explicit shared norms possible.

**Fig. 1:**
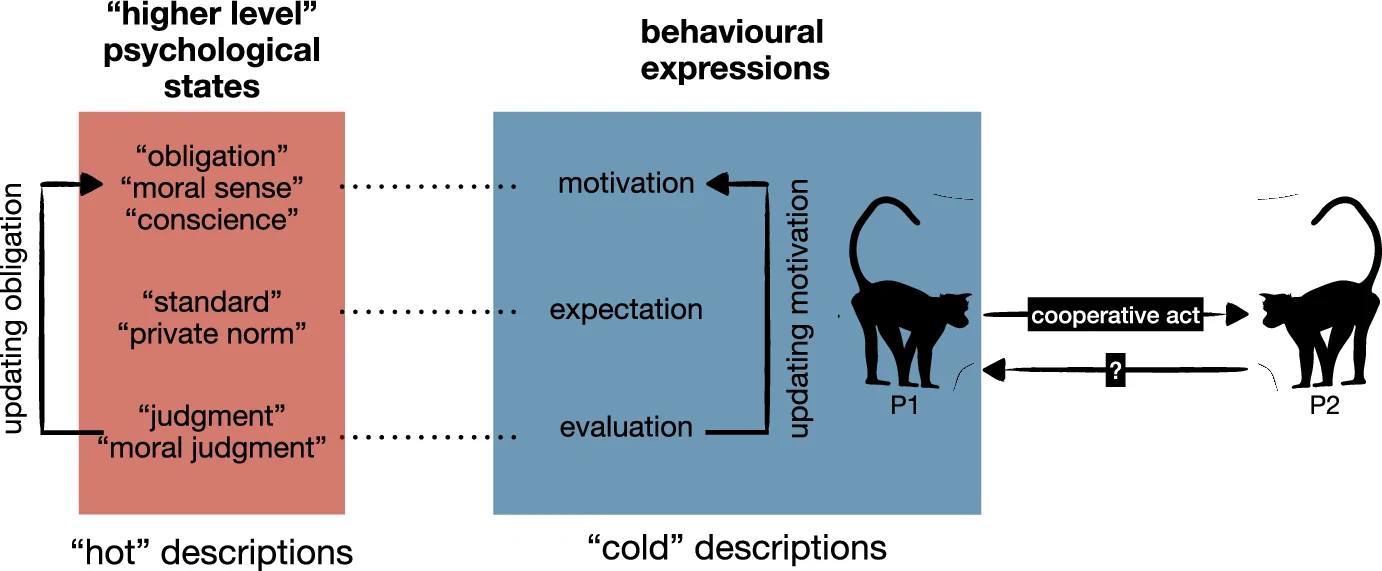
Dyadic interactions depicting the formalization of “minimal morality” and “minimal normativity”. An individual (P1) has motivations (in other words perceives “obligations”, has a “moral sense” or “conscience”) to engage in cooperative interactions and thus forms expectations (i.e., “standards” or “private norms”) that those interactions will be reciprocated. Based on the actions of the interaction partner within the dyad (P2), P1 evaluates (“judges”) and uses this information to update its motivation. “Hot” descriptions (against red background on the left) equate to the wordings usually employed by humans when talking about the higher level psychological states involved in human morality and normativity. “Cold” descriptions (against blue background on the right) equate to the wordings used when talking about the analogous behavioral expressions found in animals

This search for origins must rely on minimal definitions of these concepts, which should capture the core elements around which the other features coalesced. It can reasonably be argued that the foundational element of “the moral sense” is the presence of perceived obligations (see Fig. 1), captured in terms like “ought” (Darwin 1871) or conscience (Churchland 2019), be they proactive or reactive. In a more objective description, these perceptions reflect moral motivations (Fig. 1). Likewise, the foundational element of normativity is the emotional judging of others’ behavior in relation to some standard (Fig. 1). Behavioral violations of these expectations result in a measurable negative response, sometimes experienced as anger, and can result in updating the felt moral obligation and thus the motivation to help and cooperate (Bicchieri 2008). In a more objective description, these perceptions reflect evaluative expectations of the actions of others.

Suggestions in this direction have of course been made before, but here we want to develop them in more detail. Inevitably, we build on Darwin (1871), who already suggested that elementary morality (“a moral sense or conscience” and even a sense of duty) would be found in “any animal whatever, endowed with well-marked social instincts, the parental and filial affections being here included” but only “as soon as its intellectual powers had become as well developed, or nearly as well developed as in man” (Darwin 1871, pp. 71–72). We want to slightly amend his proposal. First, we focus on the most fundamental social relations, namely dyadic ones, in which the actor is always an interested party. Second, we claim that morality necessarily tightly coevolved with normativity. Third, this focus moves the spotlight away from the (inevitably derived) cognitive dimension (and so from questions like whether animals can be moral agents: Tomasello 2020) toward the nature and extent of helping and cooperation. Specifically, we propose that the evolutionarily oldest context of morality and normativity is to be found in relationships of one-sided dependence, namely the parent–offspring relationship, and that the same mechanisms were subsequently applied to the establishment and servicing of *dyadic interdependence relations* among species living in stable, personalized pairs or groups.

Animals can only benefit from cooperation if it is protected against exploitation. But once stable dyadic cooperation occurs, the threat of serious exploitation recedes, because partners may come to depend on each other’s help, and each therefore becomes interested in the other’s thriving. Thus, the partners’ interests (and thus fitness) become increasingly intertwined to the point that overly strong free-riding or other forms of exploitation of one’s partner becomes self-defeating because this will thwart future cooperation opportunities. The corresponding relationship of mutual help is called a social bond (Schino and Aureli 2009) and the underlying principle is interdependence (Roberts 2005). But such an evolved function requires evolved mechanisms (Tinbergen 1963). These mechanisms can be described at the psychological level in terms of motivations, preferences and the like, but also at the underlying level of neuroendocrine regulation, and (albeit convincingly perhaps only in humans) also at the level of experienced emotions or feelings (cf. Figure 1). The actors need not be aware of the functions of their motivations and preferences for those to be effective—a feature that may also at least in part apply to humans.

The establishment of dyadic social bonds essentially requires that a species lives in stable social groups that are personalized (social units in which individuals know each other), and thus small enough for individual recognition and frequent opportunities for collaboration or exchange of services. These cooperative relationships, in turn, are only viable when they are secured by two essential behavioral mechanisms: (1) a prosocial motivation to assist the other and so generate the beneficial exchanges or coordinated actions, both proactive, especially when establishing the bond, and reactive, whenever the partner needs help; and (2) a motivation to monitor and evaluate the other’s actions so as to avoid exploitation, as well as the actions of third parties to allow partner choice when previous partners disappear or partner switching if current bonds are no longer profitable.

Morality and normativity can therefore be seen as essential mechanisms through which fundamentally interdependent individuals can protect various forms of cooperation against free riding and so sustain them and harvest their fitness benefits (Darwin 1871; Trivers 1985; Krebs 2008). In the following sections, we will empirically evaluate this proposal, first for dyadic cooperation, and subsequently beyond the dyad.

### Morality and normativity in stable dyads

#### The foundation: parental care

We suggest that the evolutionary foundation of both morality and normativity can be found in parental care. Parental care requires morality: an intrinsic motivation to care for young, which must be reactive (responding to signs or signals of need), but also proactive (maintained even in the absence of direct stimuli), given that parents may not always be in direct contact with the young. The need for proactive parental motivation is especially high in parents of species with young in a nest or den, so parents must go out to forage and subsequently return to bring them food they did not ingest or digest themselves (birds: Cockburn 2006; Mock 2022; carnivorous mammals: Hudson et al. 2022) or to suckle them (mammals: (Gubernick 2013)). This urge to care is regulated by specific neuroendocrine mechanisms (Smiley et al. 2019). Separating parents from their young creates distress (expressed both behaviorally and hormonally, i.e., increase in cortisol Mendoza et al. 1978) in both parent and offspring (Bowlby 1969; Edgar et al. 2011, 2015). The same regulatory system also works in non-parents where they act as helpers, as in cooperative breeders: experimentally manipulating the need for help can produce an obligation to help (Brügger et al. 2018).

Parental care among animals also requires normativity. Parents and offspring may have largely overlapping fitness interests, and thus largely agree on investment patterns, but they naturally have a zone of conflict with respect to the intensity and duration of parental investment (Trivers 1974), which, postnatally, is expressed in behavioral conflicts triggered by parents providing a lower level of care or earlier termination of care than requested by the offspring (Clutton-Brock 1991). Both parent and offspring therefore have expectations of how the other party should behave and respond to deviations (violations) with behavioral actions, including flamboyant begging and temper-tantrums by the offspring e.g. during weaning conflicts, suggesting a strong emotional component. These expectations appear to be regulated flexibly: experiments in common marmosets have shown that when food is harder to obtain for immatures (because they lack the skills to retrieve food from a food puzzle), adults continue to share food especially proactively, but no longer share the same food if it is easy to access (Sehner et al. 2025). In a cooperative breeding system, the normative expectations also extend to helpers (alloparents), since like parents they also stop sharing food once infants reach a certain age. Importantly this drop in sharing is driven by the parents refusing to share even though immatures are still begging and not by a general decline in begging by the immatures, highlighting the conflict of the duration of investment patterns between parents and immatures (Guerreiro Martins et al. 2019; Sehner et al. 2025). These conflicts have consistent physiological correlates in both parents and offspring (Smiseth et al. 2011; Mandalaywala et al. 2014).

The proposal to found both morality and normativity in parental care is consistent with the observation that people, including children, generally feel that parenting is a moral obligation (Marshall 2020; Reddy and Wellman 2020; Xu 2021) and express normative judgments concerning parents who neglect this obligation.

#### Social bonds (friendships)

The building blocks of parental care paved the way for the evolution of social bonds, which remain fundamentally regulated in the same way (Feldman 2012; Carter 2014). Among the main proximate mechanisms assumed to be responsible for the regulation are the hormones oxytocin and vasotocin (OT-VT). These hormones were ancestrally used to regulate osmotic balance and metabolic stress (Lawson 2017; Natochin et al. 2018). These key regulators of physiological processes related to survival have subsequently evolved to facilitate physical proximity between individuals, thus creating the possibility for close bonds. Griesser et al. (2025) recently proposed that this is possible via a feedback loop that includes the activation of OT-VT release due to *touch* as a key component, which in turn facilitates social recognition as well as a decrease in antisocial behaviors, and in this way strengthens social bonds. They highlight comparative evidence from six African mole-rat species, where increased activation of the OT-VT feedback loop is found to be associated with increased touch sensitivity (Šumbera 2019; Hart et al. 2022) and cooccurs with changes in the social system ranging from solitary species (low OT levels) to eusociality (high OT levels) (Kalamatianos et al. 2010; Valesky et al. 2012; Hart and Bennett 2022).

**Fig. 2:**
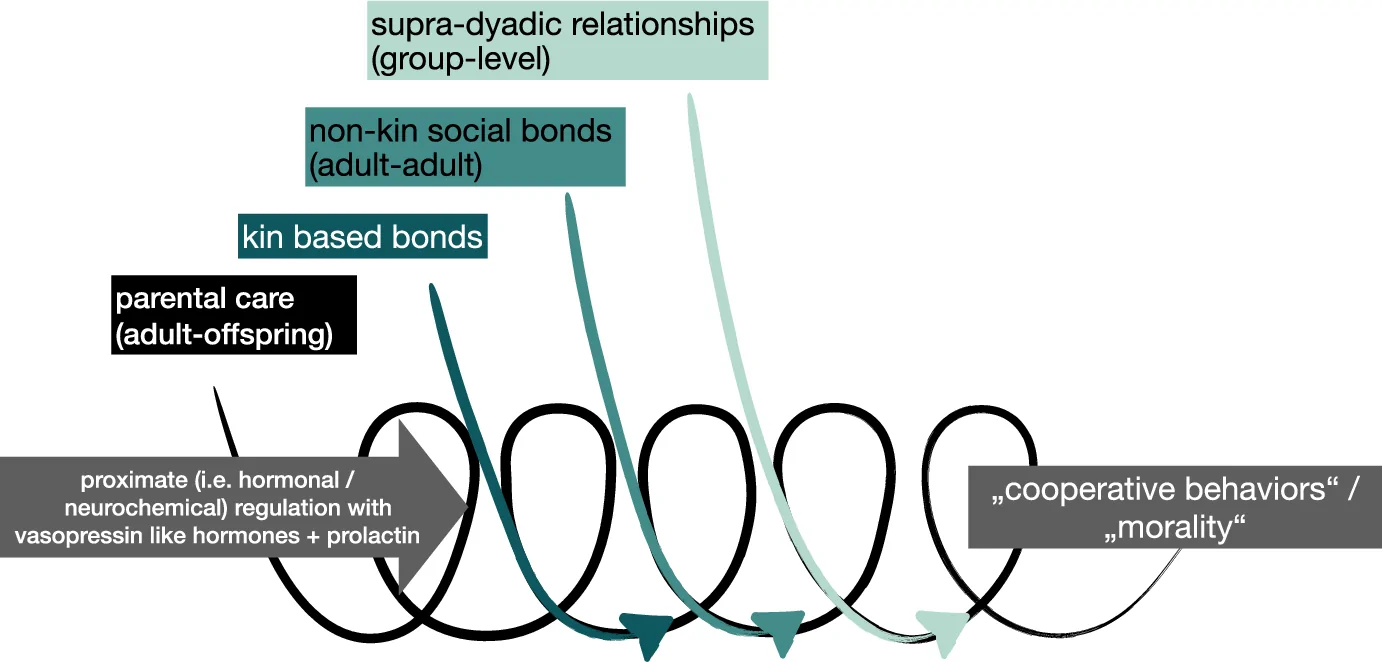
Coopting mechanisms for novel functions. The proximate mechanisms that have evolved to govern parental care (big gray arrow) are coopted in evolutionary time to govern morality/normativity in all social relationships from kin based social bonds, to non-kin social bonds to social relationships at the group level

The proximate mechanisms originally evolved for parental care are thus re-used to establish social bonds, most easily with kin, but also with non-kin and at the group level (Fig. 2). Assisting relatives is widespread and ancient in nature: biological relatedness leads to a partial alignment of fitness interests, thus limiting the benefits of free-riding and so making it easier for behavioral interdependence to become established. Kin are therefore expected to associate and form cooperative relationships more readily than non-kin, and social bonds therefore presumably first evolved among relatives. Once the regulatory mechanisms for these bonds had been fully established, relatedness was no longer required, as shown by the intense social bonds of pair-bonded partners, especially where they engage in obligate biparental care, and (phylogenetically presumably derived from this) where unrelated same-sex partners form social bonds (friendships). The latter are commonly found in species living in stable social groups that are personalized, and thus small enough for individual recognition and frequent opportunities for collaboration or exchange of services (Silk 2005, 2007; Massen et al. 2010; Sterck et al. 2024). In primates, the activation of these care mechanisms in adult-adult relationships is achieved via grooming, which is essential for the establishment and maintenance of social bonds as well as cooperation (Griesser et al. 2025).

Social bonds or friendships among kin and non-kin have been postulated since the 1970s as a positive kind of social relationship (Hinde 1976; Kummer 1978). Social bonds can be recognized by below-average rates of dyadic aggression, higher proximity, including during feeding, and the exchange of assistance behaviors such as grooming, agonistic support, or food sharing. Pair bonds are a special kind of social bond in that their interdependence primarily involves coordination of parental duties. Just like parent–offspring attachments (Bowlby 1969), both between-sex pair bonds and same-sex friendships are adaptive (Kummer 1978; Silk et al. 2009).

The concept can easily be criticized for projecting features of human friendship onto animals. However, a variety of studies have provided decisive support for the notion that the animals themselves also represent bond strength physiologically, and presumably cognitively and emotionally: bonded partners are more likely to reconcile after a conflict (Aureli et al. 2002), show partner-specific synchronized changes in oxytocin levels (Finkenwirth et al. 2015), or partner-specific increases of oxytocin in response to grooming their partner (Crockford et al. 2013; Ziegler and Crockford 2017) or to the involvement of the partner in agonistic interactions with others (Wascher et al. 2008). Like parent–offspring separation, separation from the bonded partners produces anxiety and stress responses (Mendoza and Mason 1986) and may elicit consolation behavior upon reunion (Burkett et al. 2016). In sum, although at present the detailed evidence is stronger for pair bonds than intrasexual friendships, social bonds are real for the animals too.

##### Morality and social bonds

Why do we think social bonds require morality and normativity? Social bonds involve the exchange of services (delivered either proactively or reactively in response to requests), coordinated joint actions such as coalitions, or both; pair bonds may additionally involve coordinated actions aimed at specific others: offspring. Consider the proactive side first. For social bonds to be initiated and built up, and for them to be maintained, both some proactive element and a strong reactive element are needed (the relative strength of these depends on details of the cooperation). Naturalistic observations suggest more food sharing between bonded partners in wild chimpanzees (Samuni et al. 2018). Provisioning experiments in primates generally show little evidence for proactive food offering except in cooperative breeders (Burkart et al. 2014; Marshall-Pescini et al. 2016), but where they do, food is offered selectively to bonded partners (de Waal et al. 2008; de Laat et al. 2024). This rarity may merely reflect the rarity of the establishment of new bonds in existing, stable social groups. Consistent with this idea, non-breeder common marmoset males increase their proactive food offering to novel females (Burkart et al. 2007), and there is anecdotal evidence that newly introduced captive spider monkeys show proactive sharing (J.B., unpubl.). Nevertheless, there is clearly room for targeted studies to examine the context in which proactive sharing is found.

On the other hand, extensive evidence exists for the reactive obligations (behaviorally expressed as motivation, see Fig. 1), which are key to maintaining established social bonds. Many primate studies have found that following a grooming bout, the groomee is more likely to come to the aid of the groomer than in a control situation. This has been found in various species (Seyfarth and Cheney 1984; de Waal 1997; Koyama et al. 2006; Cheney 2011; Schino and Pellegrini 2011; Tiddi et al. 2011), even in situations where both the grooming and the opportunities to provide aid were experimentally triggered (Hemelrijk 1994) or when the situation was highly abstract (Schmelz et al. 2017). These experiments demonstrate that individual *i* experiences some obligation (behaviorally expressed as motivation to reciprocate, see Fig. 1) after being groomed by fellow group member *j*.

**Fig. 3:**
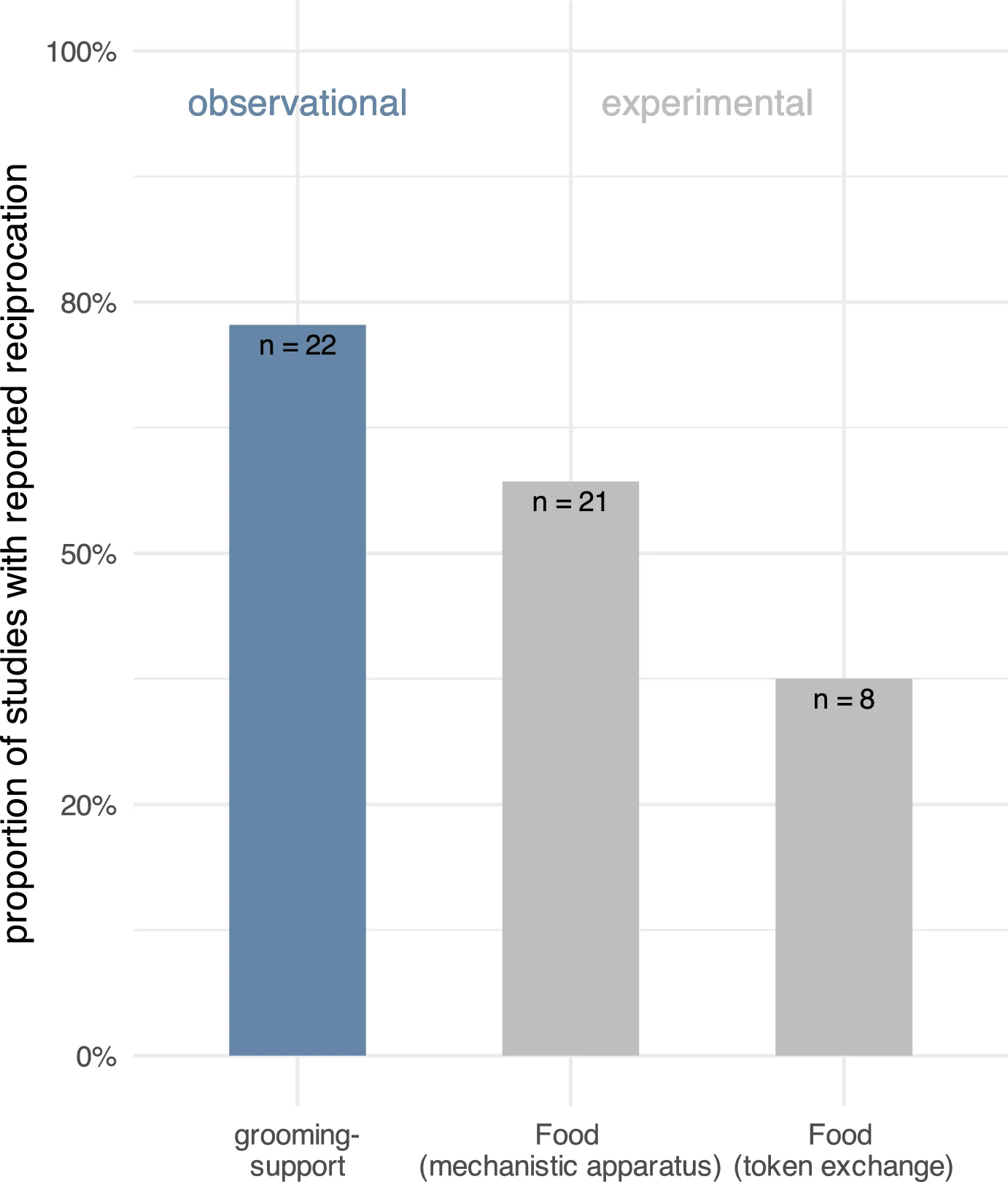
Grooming as universal „pre-payment “. Data from Schweinfurth and Call (2019) on studies investigating reciprocity in non-human primates, with percentages of studies reporting reciprocation split up between (1) observational studies where reciprocation is between grooming and support, and experimental studies where food is reciprocated via (2) a mechanistic apparatus, or (3) via token exchange. Observational studies are shown in light blue, experimental studies in light grey. N = Number of studies, p = p-value of one-sample binomial test

There even appears to be an interesting asymmetry: being paid via grooming after having received another kind of help (e.g. agonistic support) might not always work as well as receiving some help after having received grooming. Although experimental tests of this idea are of course difficult (provoking proactive agonistic support or food sharing is difficult), it might provide an additional explanation for why provisioning experiments often fail: the only proactively prosocial element in existing social bonds may be grooming (see also Crockford et al. 2013), whereas all other exchanges are reactively prosocial. This construction would also make sense of a curious pattern in research trying to establish reciprocity. Data from a review by Schweinfurth and Call (2019) show that in around 73% of experiments subjects show contingency between help received and help given when grooming was involved, versus only around half as much (38%) when the experiment involved token exchanges Fig. 3), although this difference is not statistically significant ($\chi^{2}$ = 1.8, df = 1, *p* = 0.17), perhaps reflecting the small sample size. When mechanistic apparatuses were involved and food was reciprocated via those apparatuses was intermediate. Logically, these experiments were the same, involving a quid pro quo, but the fact that most primates have trouble engaging in relaxed reciprocity when the pre-payment is not in terms of grooming (and thus involves touch) suggests a strong predisposition for the reactive prosociality to require the receipt of grooming as a trigger. That grooming and touch are particularly powerful in eliciting reciprocation of prosocial acts is strikingly in line with the above-mentioned hypothesis by Griesser et al. (2025) that states that touch, which is clearly central to any grooming interaction, is activating the hormonal feedback-loop that supports social bond formation and cooperation and that fundamentally overlaps with the proximate mechanisms of parental care (see Fig. 2).

It might alternatively be argued that bonded partners simply have more opportunities to engage in such exchanges because they groom more often. However, building up stronger obligations after receiving support from their friends than from other group members appears more likely, also in the light of results from wild chimpanzees, where grooming with bonded partners raises urinary oxytocin (regardless of the duration of the grooming bout and the direction) whereas no rise is observed when grooming is with non-bonded partners or in a control situation without grooming (Crockford et al. 2013).

##### Normativity and social bonds

Effective social bonds not only require strong prosocial motivations, which can be experienced as obligations, but also the second mechanism, social evaluation. Monitoring and some means of controlling the behavior of the partners (partner control: Noë and Hammerstein 1994; West et al. 2007; Johnstone and Bshary 2008; Barclay 2016; Schino and Aureli 2017) is needed because the fitness interests of partners are never perfectly aligned and some temptation to shirk or free-ride is always present, even among kin (Bshary et al. 2016). This monitoring comes in the form of observing and evaluating the social actions of partners, primarily as experienced directly in social interactions with them, but secondarily also as observed in their social interactions with others. Deviations from expectations trigger responses, ranging from reduced willingness to respond to requests for assistance to shunning or even active partner control in the form of punishment.

Empirical evidence that social animals use this information to sanction ‘violations’ by bond partners is less common here because the most common response to unfair treatment by bond partners is to shun them and switch to another one (Noë and Hammerstein 1994). Thus, active punishment is rather rare, but where it occurs it effectively restores cooperation (Raihani et al. 2010, 2012). This rarity may reflect the special conditions in which it is expected: one partner is more powerful than the other, but both benefit from continuing cooperation rather than partner switching. For example, cleaner fish males punish females for ‘cheating’ the clients by feeding on mucus rather than ectoparasites but can do so only because they are clearly bigger.

An interesting exception concerns the context when the two partners must work together to obtain a reward, such as simultaneously pulling the two ends of a rope looped around a food platform to acquire the reward (e.g., Crawford 1937; Suchak et al. 2016). Here, we sometimes do see agonistic responses to shirking, probably because without these responses there is no benefit and because to get coordinated collaboration going in the first place requires a high level of social tolerance.

As a result of these difficulties, the most abundant evidence for evaluation of the actions of bond partners comes from a different setting: the relationship between experimenters and captive primates. Following Brosnan and de Waal (2003), many experiments were done in which an experimenter dispensed food rewards of contrasting quality (e.g. cucumber versus grape) to two subjects, with or without ‘work’ (receiving and returning a token to obtain the food). In the experiment, one subject received lower-quality food than another group member next to it, and its responses were recorded. What exactly is measured by this paradigm has been subject to much debate (Bräuer and Hanus 2012; Brosnan and de Waal 2014; for a recent meta-analysis see Ritov et al. 2024), but probably the best-fitting explanation, confirmed in chimpanzees (Engelmann et al. 2017), is that the experiment is testing the bond between experimenter and subject, rather than the notion of inequity aversion (in which subjects compare the way they are treated with how other subjects are treated). The protests by subjects that received low-value food rewards can therefore be seen as responses to inadequate reciprocation by bonded partners (here: the experimenter) rather than to general, group-wide norms, as in fairness. This interpretation is supported by a common experience in cognitive tests that involve rewarding subjects for correct responses: when subjects fail and thus are not rewarded, they may vent their dissatisfaction by threatening the experimenter (R.B. & J.B., pers. obs.).

#### Morality and normativity beyond dyads

So far, we have examined only dyads. Thus, the moral urges and social evaluations always referred to self and partner only. However, individuals may also monitor non-bond members when current bonded partners disappear or for whatever reason lose their social utility. The presence of dyad-level social obligations and monitoring could therefore serve as the foundation for group-level obligations when the adaptive need for this arises and all dyads uniformly develop strong bonds. Indeed, when we think of morality and normativity in humans, we think of obligations and norms shared by all or most members of a society or a local culture, not *just* an individual, and an awareness among all or most group members of this shared status.

Among animals, such sharing at the group level is likely to be rare, since the potential for individual free riders or exploiters will have to be kept in check by the joint action of the majority. We are thus looking for situations in which interdependence among individuals involves more than two individuals or is even group wide. These conditions can be met when (1) preferences are shared by all: group-wide norms (“social norms”) become emergent properties as soon as violations to private norms are shared and lead to joint protests when any individual of the group violates this shared preference; and (2) when high-risk but high-benefit coordinated collective action is required that involves many individuals. In those situations, partner monitoring is expected to be intense and thus sanctions (individual or joint) against non-participants may be strong. These situations are the precursors to the far more elaborate group-wide or species-wide moral norms seen in humans.

A critical precondition for this is that animals perceive social interactions between third parties holistically and with reference to bond strength, rather than as a mere series of actions without taking the dyadic context into account. An elegant experiment by Bergman et al. (2003) supports this assumption: when a series of playbacks contained the friendly approach grunt of a lower-ranking baboon females followed by the submission shriek of a higher-ranking female, bystanders responded with longer looking times, as if surprised. Likewise, Brügger et al. (2021) used thermography of the nasal region to show that common marmosets perceive a series of vocal signals between two conspecifics as a whole, rather than as a mere series of calls.

This perceptive ability may also allow individuals to respond to third-party violations of dyadic standards because they might be affected similarly in the future and thus may reduce future risk to themselves by showing solidarity. The most common of such situations concerns infanticide by males. In virtually all cases described in the literature, the attacks are greeted by an explosion of calls suggesting a mix of alarm, protest and aggression (van Schaik and Janson 2000) by the majority of group members. This may look like a shared norm by most group members (although obviously not by the perpetrator who stands to gain from his act). Yet, despite this case of convergent dyadic norms, there are only very few other known examples (Burkart et al. 2018). For instance, we know of no reports of other group members responding to infant abuse by other females, or of reactions to observing that a bonded partner fails to come to the aid of a friend who recently groomed them.

This raises the question why individuals do not regularly extend their motivations and expectations concerning the behavior of others to all other group members and thus do not respond to the violations of their own standards when they observe them as uninvolved bystanders. If they did, this would reflect true group-level standards; after all, most individuals are expected to share the same social standards. It may even be adaptive, in that a group in which conflicts remain low will do better and thus may benefit from group augmentation effects. One reason for their seeming absence may be that interfering in conflicts among others may involve risk (especially in groups with a steep dominance hierarchy where the violations are committed by high-ranking individuals). Another one is that selection may not favor such interference in the interactions of others because the benefits of improved group performance are close to public goods (tend to be shared rather equally), leading to a secondary collective action problem (Panchanathan and Boyd 2004).

Improved prospects of reaping benefits from social bonds may also produce a tendency toward conformity among animals, reflecting convergent preference for behavioral similarity in others. Immigrants in cooperative breeders provide infant-care to an extent indistinguishable from resident helpers who are related to the infants (Laubi et al. in prep). In other species, immigrants abandon existing feeding preferences if the host group’s preferences differ (van de Waal et al. 2013; Luncz and Boesch 2014). Future work should show whether the normative element is strong enough to show that those who do not adopt the residents’ norms are punished (but see van de Waal et al. 2013, for a possible case).

Overall, then, there is rather scant evidence among primates that individuals impose norms on others. Indeed, existing group-level standards may often be the byproduct of convergent individual responses. True group-level standards should therefore be more likely as group members become more interdependent (and thus share moral preferences) and where the costs of interfering with transgressions are low. Group-wide interdependence is expected in species with widely shared cooperative tasks for which coordination is essential, as in cooperative breeders. Interference should be more likely where it produces beneficial reputational benefits, or where groups are more egalitarian, and individuals or alliances of lower-rankers may effectively contain high-rankers. These two conditions obviously apply to human foragers (e.g., Boehm 1999; Pandit and van Schaik 2003). However, some of the known animal cases may also fit these conditions. Best known are peaceful interventions in conflicts among chimpanzees (de Waal 2007). A comparative analysis involving multiple groups could show that these interventions are most likely in conflicts that might otherwise escalate, and during periods of social instability, for instance due to immigration. The arbiters were usually old females or high-ranking males, with a tendency for males of all ranks and ages to be more involved (suggesting a female preference for such males) (Rudolf von Rohr et al. 2012; de Waal 2014).

Humans are unique in this respect, most likely because the highly interdependent lifestyle of the foraging niche of hunter gatherers necessitated this as an essential psychological foundation for this lifestyle (Tomasello et al. 2012). There is abundant evidence for group-level moral preferences and norms. Thus, sharing patterns are not only restricted to specific dyads but community wide; free riding, even by small numbers of group members, would have repercussions for all group members. This in turn makes all group members highly motivated to ensure that others do not violate the “sharing norm.” The group-wide sharing that is universal among human foragers (Gurven 2004; Jaeggi and Gurven 2013; Dyble et al. 2016) is taxonomically rare, perhaps because gossip helps to create the consensus needed for collective punishment. This would thus explain why group-level morality and normativity is concomitantly rare among animals.

In fact, humans have moral norms that go even beyond their own group or tribe. Since times immemorial, forager groups have exchanged precious items like obsidian knives (Ortega et al. 2014; Ibáñez et al. 2015) or had mutual sharing arrangements with other groups in times of famine (Wiessner 1982). This interdependence in space and time beyond the current group may underlie the intuition that led philosophers to formulate universal moral norms. The phenomenon and the broad principles to which it gave rise depend on the presence of language and the ability to formulate and negotiate binding principles at larger social scales.

Awareness of being observed and evaluated by others is not necessary for the presence of group-wide moral norms to work. It is, however, expected when the requisite cognitive resources are available because it improves a subject’s chances of being selected as a cooperation partner or mate. Moreover, it is all but required in contexts with reputation-based indirect reciprocity and a critical precondition for shared intentionality. Such reputation management has a mixed record among primates. There is no audience effect, for instance, on chimpanzee food sharing in captivity (Engelmann et al. 2012). However, Hector et al. (1989) reported that male vervet monkeys were kinder to infants when their mother could observe (but not intervene in) their interactions. Immature monkeys and apes self-handicap when playing with others whenever these are close to their mothers, who could potentially intervene (Palagi 2018). Male common marmosets respond differently to an unfamiliar female when their female mate is present or not (Evans 1983). These examples suggest that where such reputation management is adaptive, animals have acquired it, suggesting its taxonomic distribution is not limited by strong cognitive constraints.

## Conclusions

In this paper we showed how morality and normativity are phylogenetically old and taxonomically widespread, arose in dependence (parent-offspring) relationships and were coopted for interdependence relationships (socially bonded group members). Our reconstruction also requires that morality and normativity inevitably go together, and that they are originally social. Thus, each species with interdependency relationships is expected to have an adaptive morality and normativity, whose depth and reach depend on the number and structure of these interdependencies within and between social units. Since the behavioral expressions of the cooperative interactions involved in minimal morality and minimal normativity originated in parental care, only those behaviors present can be coopted to service interdependence relationships. Consequentially if the behavioral repertoire of infant care in a certain species is larger, the potential behavioral expressions of actions that are experienced as obligations and thus are “moral” in nature is wider, this might apply especially in species with cooperative infant care such as humans or callitrichid monkeys.

Human morality and normativity are not fundamentally different, but in our species owing to our greater interdependence at multiple levels, their reach extends well beyond the dyad to encompass entire social units, with a tendency to extend beyond the local social unit. Obviously, language, built on shared intentionality (Tomasello et al. 2005, 2012), allows for the explicit formulation of norms and of responses to norm violation, which also explains the cultural variability of human morality and norms (Roughley and Bayertz 2019). Language as a powerful engine to allow for moral externalism (i.e. being able to make statements about universal and externally imposed norms, Stanford 2018) maybe via the mechanism of externalized commitment (Khan 2025) could be a strong mechanism to secure human hyper-cooperation. The major social changes following the origin of sedentism and food production added various social structures that invited derived moral norms beyond the original care/harm and the fairness/cheating dimensions (Haidt 2013).

We have explicitly argued for social origins of morality and normativity. Some doubt has been expressed about whether the origin of normativity in particular lies in the social domain (Korsgaard 1996). However, there is ample evidence in humans that our perception of causal events in the world is highly biased towards inferring agency, i.e., causal chains are interpreted as an agent being responsible for them, our very own perception is thus agent oriented (van Schaik 2016, see also Thomas 2024). We know that nonhuman primates are also searching for agents when interpreting the world (Burkart et al. 2012; Kupferberg et al. 2013), and that human–machine interactions are strongly affected by how agent-like the machine is (Abubshait and Wiese 2017). Thus, a social origin of morality is highly plausible, and this social bias probably affects how we think about moral (and normative) arguments that are applied to non-agent situations. Likewise, it is sometimes argued that moral judgments are made because humans are highly cognitive and reflective, i.e. morality is just a necessary consequence of the cognitive abilities of humans and not an adaptation itself (Machery and Mallon 2010; Ayala 2010). Our account, on the other hand, provided many arguments as to why moral arguments originated in social contexts, even though they can be extended to non-social contexts as well.
